# Supplementary material for: Zinc-Dependent Oligomerization of Thermus thermophilus Trigger Factor Chaperone
Source: Biology (Basel). 2021 Oct 26;10(11):1106. doi: 10.3390/biology10111106 (PMC8614707; doi:10.3390/biology10111106)
Supplement: Supplementary file 1 [file biology-10-01106-s001.zip › biology-1415786-supplementary.pdf]

Supplementary Materials for

Zinc-dependent oligomerization of *Thermus thermophilus* trigger factor

Haojie Zhu, Motonori Matsusaki, Taiga Sugawara, Koichiro Ishimori*, and Tomohide Saio*

* Correspondence: saio@tokushima-u.ac.jp; Tel. +81-88-634-6412

koichiro@sci.hokudai.ac.jp; Tel. +81-11-706-2707

This PDF file includes:

Table S1

Figs. S1 to S4

Table S1. The primers and templates for plasmid construction.

Primer	Oligo sequence (5' to 3')	PCR template	Constructed plasmid
1	CATCACGTGGCGGAGATCCTG	*Tt*TF gene segment	*His$_6$*-*Tt*TF
2	GAACAGAACTTCCAGTCAGCCCAGGG		
3	CCGCCACGTGATGATGATGAT	pCold *Ec*TF^{RBD} vector	
4	TGACTGGAAGTTCTGTTC		
5	CATCATCACATGGAAGTTAAAGAACTTG	*Tm*TF gene segment	His$_6$-*Tm*TF
6	CCGCGGATTAGTTGTCCTCCTTCCC		
7	GACAACTAATCCGCGGGTCTGGAA	pCold *Ec*TF^{RBD} vector	
8	AACTTCCATGTGATGATGATGATGATG		
9	GAAAACCTGTATTTTCAGGGTGTGGCGGAGATCCTGGAG	His$_6$-*Tt*TF	His$_6$-TEV-*Tt*TF
10	ACCCTGAAAATAAGGTTTTCGTGATGATGATGATG		
11	GAAAACCTGTATTTTCAGATGCAAGTTTCAGTTGA	His$_6$-*Ec*TF	His$_6$-TEV-*Ec*TF
12	CTGAAAATACAGGTTTTCGTGATGATGATGATGATG		
13	CCCCGAGTGACTGGAAGTTCTGTTC	His$_6$-TEV-*Tt*TF	His$_6$-TEV-*Tt*TF^{RBD}
14	TCCAGTCACTCGGGGTAGTTCTCCAC		
15	TCAGGGTCCCGAGGTGAGGCTTCCC	His$_6$-TEV-*Tt*TF	His$_6$-TEV-*Tt*TF$^{PPD-SBD}$
16	ACCTCGGGACCCTGAAAATACAGGTT		
17	CTACGCCGAGCTGGACGAGGAGTTC	His$_6$-TEV-*Tt*TF$^{PPD-SBD}$	His$_6$-TEV-*Tt*TF^{SBD}
18	TCCAGCTCGGCGTAGCGCTGGCGGAG		
19	GGTAGTGGCAGCGGAGAGCTGGACGAGGAGTTC	His$_6$-TEV-*Tt*TF^{SBD}	His$_6$-TEV-*Tt*TF^{SBD} (GSGSG)
20	TCCGCTGCCACTACCGGCGTAGCGCTGGCGGAG		
21	TCAGGGTGCCGAGCTCGTCCCCGTG	His$_6$-TEV-*Tt*TF	His$_6$-TEV-*Tt*TF^{PPD} (step 1)
22	AGCTCGGCACCCTGAAAATACAGGTT		
23	CCCCGAGTGACTGGAAGTTCTGTTC	His$_6$-TEV-*Tt*TF^{PPD} (step 1)	His$_6$-TEV-*Tt*TF^{PPD}
24	TCCAGTCACTCGGGGAGCTTCAGGGT		
17	CTACGCCGAGCTGGACGAGGAGTTC	His$_6$-TEV-*Tt*TF	His$_6$-TEV-*Tt*TF$^{RBD-SBD}$
18	TCCAGCTCGGCGTAGCGCTGGCGGAG		
19	GGTAGTGGCAGCGGAGAGCTGGACGAGGAGTTC	His$_6$-TEV-*Tt*TF$^{RBD-SBD}$	His$_6$-TEV-*Tt*TF$^{RBD-SBD}$ (GSGSG)
20	TCCGCTGCCACTACCGGCGTAGCGCTGGCGGAG		

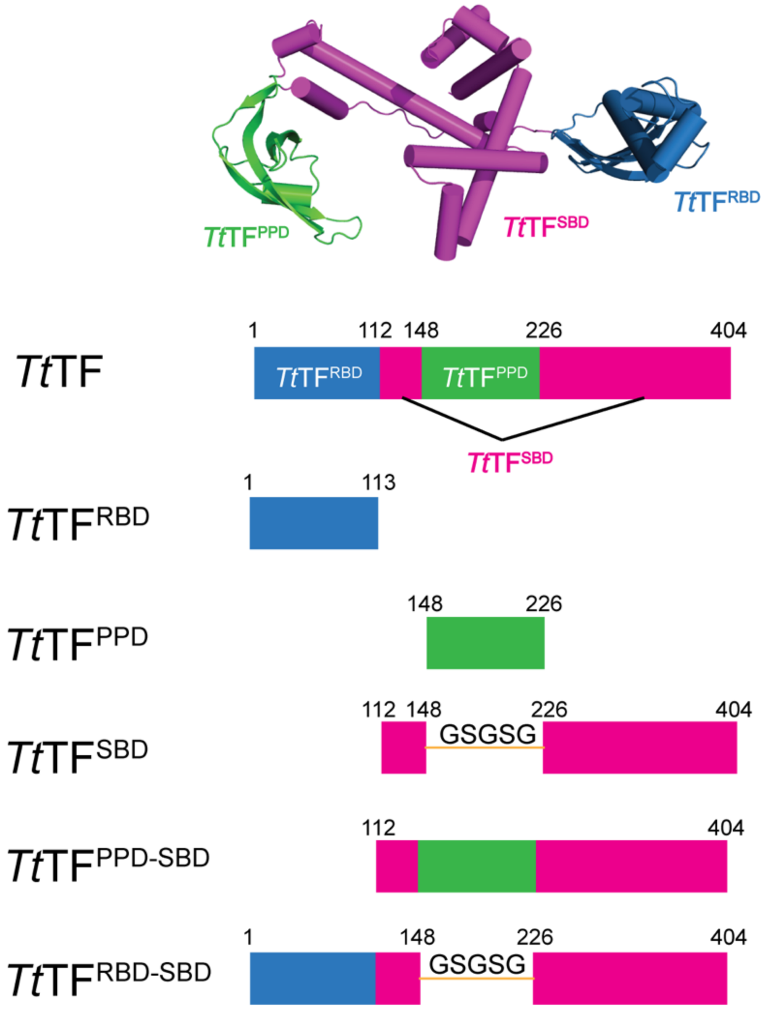

Figure S1. The domain architecture of *Tt*TF. The domain architecture of *Tt*TF and the constructs used in MALDI-TOF-MS experiments are shown with predicted structure of *Tt*TF . The coordinate of *Tt*TF was calculated by Alphafold2 (https://colab.research.google.com/drive/1LVPSOf4L502F21RWBmYJJYYLDlOU2NTL, Accessed date: 17 August 2021).

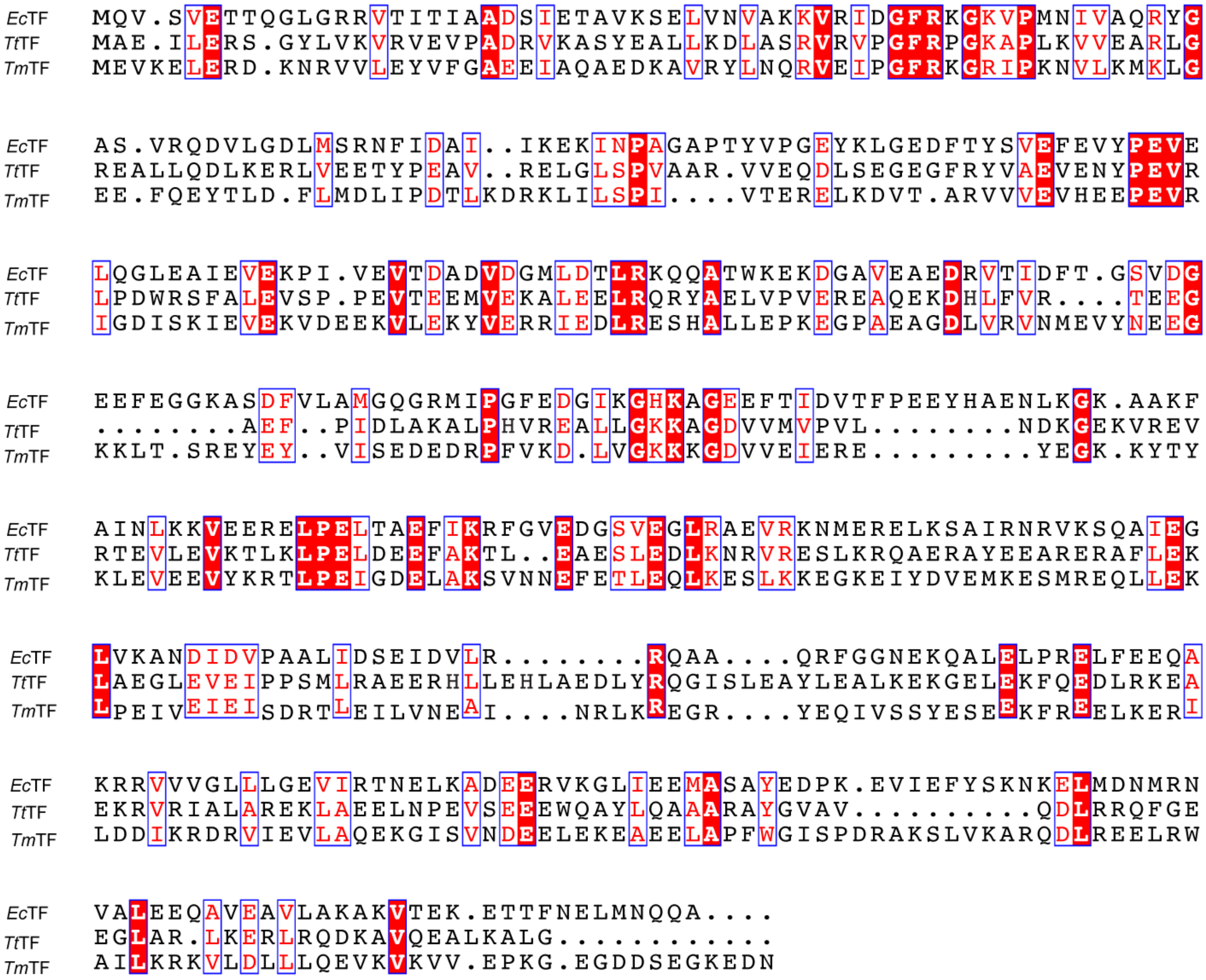

Figure S2. Amino acid sequence alignment of *Ec*TF, *Tt*TF, and *Tm*TF. The conserved amino acid residues are shown in blue frame and the identical residues are highlighted in red background. The identity and similarity between *Ec*TF and *Tt*TF are 26% and 49%, respectively. The identity and similarity between *Tt*TF and *Tm*TF are 35% and 52%, respectively. The identity and similarity between *Ec*TF and *Tm*TF are 24% and 45%, respectively. The sequence alignment was performed operated on the BLAST web server (https://blast.ncbi.nlm.nih.gov/Blast.cgi, Accessed date:14 April 2021, and 9 October 2021).

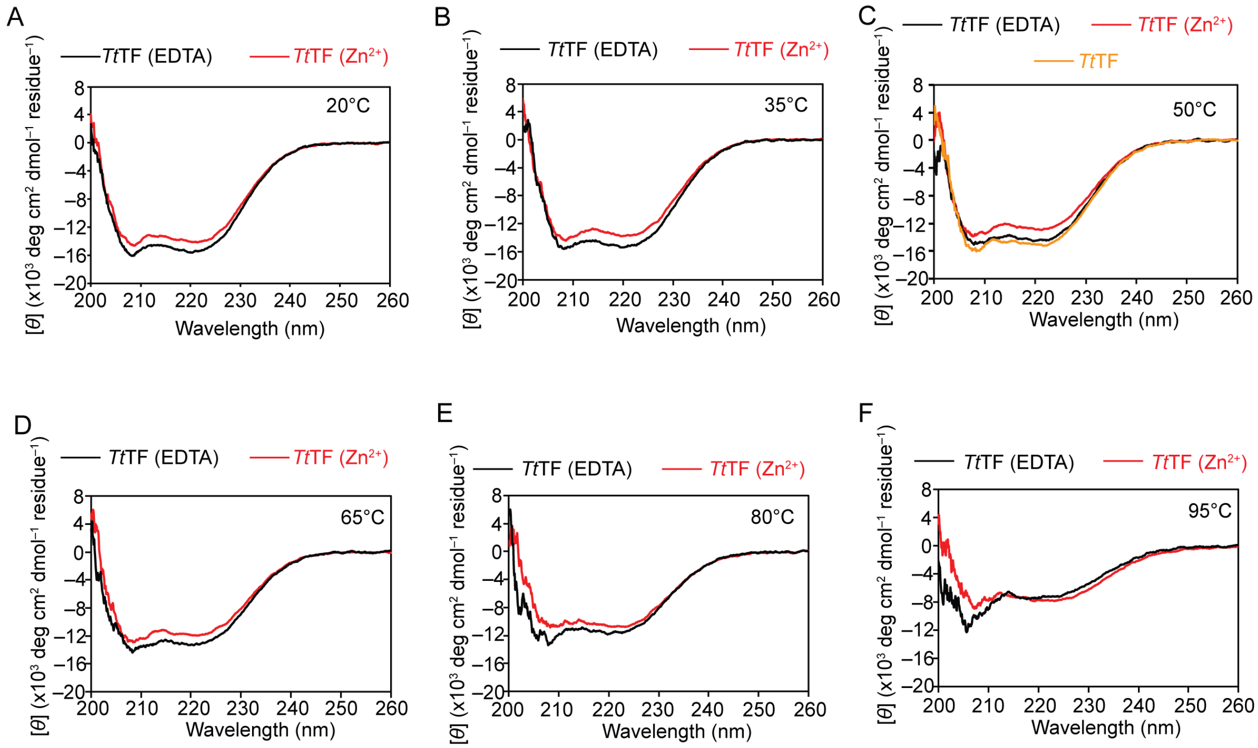

Figure S3. The CD spectra of *Tt*TF at varying temperatures. The CD spectra of *Tt*TF (Zn^{2+}) (red) and *Tt*TF (EDTA) (black) at 20°C (A), 35°C (B), 65°C (C), 80°C (D), and 95°C (E) from 200 nm to 260 nm are displayed. The CD spectrum of *Tt*TF before refolding at 50°C (orange) (C) is superimposed, showing that the spectrum of *Tt*TF before refolding is essentially the same as that of *Tt*TF (EDTA).

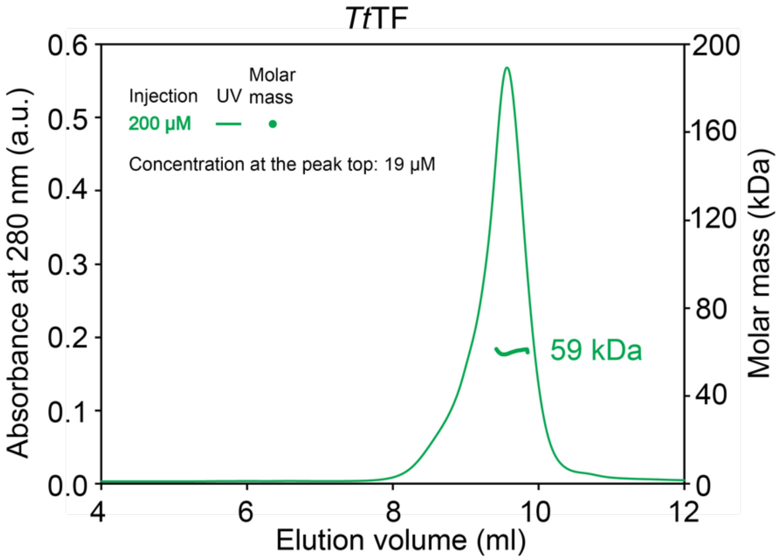

Figure S4. SEC-MALS profile of the natively purified *Tt*TF before refolding. The mass plots show that *Tt*TF exists mostly as a monomer at ~19 μM, which coincides with the SEC-MALS data of *Tt*TF (EDTA).
